# Sex-related differences in experimental pain sensitivity in subjects with painful or painless neuropathy after surgical repair of traumatic nerve injuries

**DOI:** 10.1097/PR9.0000000000001033

**Published:** 2022-10-20

**Authors:** Adriana Ana Miclescu, Panagiota Gkatziani, Pontus Granlund, Stephen Butler, Torsten Gordh

**Affiliations:** Department of Surgical Science, Uppsala University, Uppsala, Sweden

**Keywords:** Chronic pain, Peripheral neuropathic pain, Experimental pain, Endogenous pain inhibition

## Abstract

**Introduction:**

Sex-related influences represent a contributor to greater pain sensitivity and have a higher prevalence of many chronic pain conditions, including neuropathic pain (NP), among women.

**Objectives:**

The aim was to analyze how differences in ongoing pain, experimental pain intensity, and conditioned pain modulation (CPM) relate to sex in subjects with neuropathy after traumatic nerve injuries.

**Methods:**

Endogenous pain modulation was compared between male (n = 77) and female (n = 55) subjects and between subjects with NP (female = 31, male = 39) and pain-free subjects with posttraumatic neuropathy (female = 24, male = 38). Conditioned pain modulation was assessed by pain ratings to pressure stimuli before and after a noxious conditioning stimulus (CS) conducted with one arm submerged in cold water (4°C) for 1 minute. Time of recovery (Time off) of pain intensity from peak VAS_maxc_ after CS was recorded and compared between male and female patients.

**Results:**

Greater ongoing pain intensity was found among female patients compared with male patients and more experimental pain after pressure and cold induced pain. Summing all groups together, women had 0.8 times higher odds (20%) of recovering sooner than men after CS (95% CI = 0.65–2.9). No differences in CPM, time off, and psychosocial variables were seen between female and male patients (*P* < 0.05).

**Conclusion:**

Our hypothesis for sex differences in endogenous pain modulation was only supported by a shorter after-sensation time after cold CS in female patients. No sex differences in the magnitude of CPM effect were identified. Increased pain intensity for experimental pain, in both neuropathic pain and neuropathy without pain, was found in female patients.

## Abstract 2

Higher pain intensities at all experimental stimuli but a tendency to faster recovery after cold conditioning stimuli were seen in women with neuropathy in comparison with men.

## 1. Introduction

Population-based research suggests a higher risk of several pain conditions,^16,49,51^ including chronic neuropathic pain,^10,46^ in women compared with men. Chronic neuropathic pain patients are heterogeneous with a multitude of confounding genetic, pathophysiological, and psychosocial elements, all of which are variably expressed in each individual. The influence of sex hormones in neuropathic pain is receiving increased attention because it represents a significant source of pain-related variability.^1^ The current consensus is that estrogen reduces hyperpolarization postinjury of the peripheral nerve system and acts in a both preventive and hyperalgesic manner, whereas male hormones have antinociceptive effects.^27^ Attention has been also focused on the action of sex hormones on endogenous pain modulation as a contributor to greater pain sensitivity and higher prevalence of many chronic pain conditions in women.^36^ For instance, in some studies, male patients expressed more effective descending inhibition quantified by conditioned pain modulation (CPM).^7,8,13,34,39^ In other studies, no sex differences^3,52^ or more CPM reproducibility/stability in women with chronic pain were seen.^29^ This ambiguity may be because of different pain conditions studied and different testing paradigms used.

We have previously demonstrated that CPM cannot be used as a mechanism to differentiate chronic neuropathic pain from neuropathy without pain because the neuropathic pain patients after trauma and surgery had well-functioning CPM, similar to that of patients with the same type of nerve lesions but with no pain.^30^ In this follow-up study, we hypothesized that sex differences would be reflected in different endogenous inhibitory systems that would confer risk for or protection against persistent pain. Thus, we intended to find (1) whether men and women with painful vs posttraumatic painless neuropathy are similarly sensitive to experimental stimuli and (2) whether they have similar CPM effects. Secondary analysis examined the differences between men and women in (1) pain severity after a standardized stimulus, (2) psychological factors as anxiety and depression, and (3) the degree of pain-related disability. The answers to these questions represent priority areas for understanding and treatment of pain conditions.

## 2. Methods

This study has been carried out at the Multidisciplinary Pain Center, Uppsala University Hospital, (UUH), Sweden. The study followed the ethical guidelines from the Declaration of Helsinki and was approved by the Regional Ethics Committee (Project identity: ICONSS, Dnr: 2015/265; registered at www.ClinicalTrials.gov NCT03174665). Informed consent was obtained from all participants.

### 2.1. Participants

All participants of the study were recruited from a larger cohort of more than 1000 patients with traumatic nerve lesion in the hand or lower arm region, admitted (between 2009 and 2015) at the Hand Surgery Clinic, UUH. In January 2016, questionnaires about pain intensity, previous medication, and the Self-Administered Leeds Assessment of Neuropathic Signs and Symptoms (S-LANSS questionnaire) were sent to all the subjects. Of these, 706 patients returned the questionnaire (response rate of 67.1%). Thirty-seven patients without nerve injury (n = 8) or with no indication for surgery (n = 29) were excluded. Of the remaining 669 patients, 50.3% (n = 337; men n = 248, women n = 89) experienced chronic postsurgical pain and 332 patients were pain free (men n = 243, women n = 89).^31^ Nerve suture (neurorrhaphy) was the most common procedure (79.3%). Among the patients who underwent nerve suture surgery were 271 patients with persistent pain (men n = 199) and 260 patients (women n = 194) without pain. According to their answers to the questionnaires, a group of patients with pain and a group of pain-free controls were invited to participate in a follow-up study.^30^ The inclusion criteria for the participants were age ≥18 years, a surgical nerve repair after a traumatic nerve lesion in hand and lower arm region, chronic postoperative neuropathic pain with a history of 6 months to 7 years before the screening visit, pain intensity more than 50 mm on a 100-mm visual analogue scale (VAS) for the group of the participants with pain, and intensity of pain <20 mm VAS for the group without pain. The exclusion criteria were evidence or history of any clinically significant neurological disease or other systemic diseases or conditions potentially interfering with study assessments (polyneuropathy, diabetes mellitus, peripheral vascular disease, history of malignant disease, or chronic alcohol consumption).

All the participants were prescreened by the Self-report Leeds Assessment of Neuropathic Signs and Symptoms (S-LANSS questionnaire).^30^ The participants with pain and S-LANSS ≥12 (indicating predominantly neuropathic pain)^5^ were recruited for the group with neuropathic pain and those with S-LANSS <12 and sensory impairment were recruited for the group with neuropathy without pain.

### 2.2. Eligibility for participants

Seventy-three subjects (34 women and 39 men) with pain and 73 subjects (34 women and 39 men) without pain were examined. Biological sex (male/female) was reported because patients were not asked to self-report. The confirming sensory impairment on examination of the somatosensory system with pain in the innervation territory of a previous intraoperatively verified injured nerve strongly indicated a diagnosis of “definite neuropathic pain” for all the subjects with pain.^11,48^ All the subjects had a definite traumatic nerve lesion, seen by the surgeon intraoperatively. Eligibility for participants was determined only after completion of a health history questionnaire, interview about pain intensity, and a routine clinical neurological examination. All participants were asked to refrain from any pain medication for at least 12 hours before the experimental session.

### 2.3. Procedures

All participants were informed about the test program before (by telephone) and after arrival at the laboratory. Participants attended a single appointment. A standardized case report form was used to collect a detailed pain history that included type of pain, duration and its characteristics, and current and previous pharmacological and nonpharmacological pain management. Sociodemographic data included education level, work status, and family and medical history. Body mass index was calculated using the formula weight/height^2^ (kg/m^2^). Baseline brachial resting blood pressure was examined before the experiment was started. All sessions followed the same procedure and were performed by the same trained examiner who read from a standardized instruction protocol when performing CPM.

#### 2.3.1. Pain assessment and clinical examination

After clinical evaluation and additional tests (nerve conduction studies before the visit, QST), pain was classified as neuropathic or nonneuropathic using the International Association for the Study of Pain/Neuropathic Pain Special Interest Group grading system.^11^ Sensory examination testing was for touch with a camel-hair brush (0.5 Somedic, Sweden), pain with a sharp tooth pick, and temperature with warm (40°C) and cold (25°C) rollers (Senselab Rolltemp, Somedic). The contralateral uninjured side served as within subject control. Participants were asked to rate their mean clinical pain over the past week on VAS (0–100).

#### 2.3.2. Conditioned pain modulation

**Figure 1.**
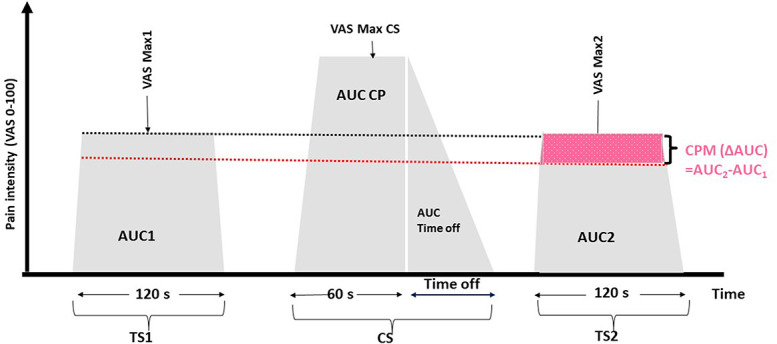
Timeline showing CPM stimuli administration. TS, test stimulus (pressure pain); CS, conditioning stimulus (cold water); TS1, pressure pain ratings during the first test stimulus (120 seconds) and return to baseline; TS2, pressure pain ratings during the second test stimulus (120 seconds) and return to baseline; CS, pain ratings during conditioning stimulus (60 seconds) and return to baseline; Time off, time to return to baseline after CS; AUC1, area under the curve calculated from pain rating over time during TS1; AUCCS, area under the curve during CS; AUC2, area under the curve calculated from pain ratings over time during TS2.

The CPM paradigm that has been described previously involved a tourniquet pressure test stimulus (TS) applied to one leg before and after a thermal conditioning stimulus (CS) by immersion of the uninjured hand in 4°C cold water (Fig. 1).

#### 2.3.3. Test stimulus

The TS was delivered by a tourniquet applied mid-calf on the leg corresponding to the noninjured arm and inflated from 60 to 100 mm Hg above the systolic blood pressure until the pain intensity (typically 220–250 mm Hg) reported by the subject was over 50 on a 0 to 100 visual analogue scale (VAS). The test stimulus (TS) was applied for a duration of 120 seconds before (TS1) and after (TS2) the conditioning stimulus (CS) at the same pressure.

#### 2.3.4. Conditioning stimulus

The conditioning stimuli was given by having subjects immerse their noninjured hand up to the wrist in a cold-water bath at 4°C cooled by a refrigerated water circulator (Somedic, 2015, Sweden) for a maximum of 1 minute. The CS was applied immediately after the subject became pain free after TS1 and ended when the subject withdrew the hand from the cold-water bath or maximally for one minute. The water level was set at a height of 30 cm and maintained at a constant temperature to keep the stimulated area consistent. Time in the cold-water bath (Time CS) and time until the pain intensity decreased and the subjects became pain free (Time off or time after sensation) after removing the hand from cold water were expressed by the area under the curve (AUC_CS_, AUC_time off_).

Immediately after the subjects became pain free after the conditioning stimulus, an identical test stimulus (TS2) was repeated. The subject was instructed to rate continuously the pain intensity level of both the test stimulus and the conditioning stimulus with the eVAS slider until they became pain free. They could terminate the trial at any time if they could not tolerate the painful pressure (120 seconds) or cold water (60 seconds).

#### 2.3.5. Conditioned pain modulation

To quantify CPM, the deviation of pain ratings from the set point was continuously recorded and summed over time to produce an area under the curve (AUC) value. From the starting point of the first test stimulus forward, this dependent variable (AUC) of the VAS response over time was calculated for both the test stimuli (AUC_1_ and AUC_2_) and conditioning stimulus (AUC_CS_). Thus, CPM was calculated as the difference in area under the curve of pain rating responses between the last test stimulus after CS and the test stimulus before CS (∆AUC = AUC_2_ − AUC_1_).

The CPM effect (%CPM) is defined as the percent change of the pain intensity evoked by TS induced before and after CS. The formula used for CPM effect change is as follows: [(TS2 pain − TS1pain)/TS1 pain] × 100. The percentage of CPM (%CPM) = ∆AUC × 100/AUC_1_. The CPM effect varies from pain inhibition to facilitation. Therefore, negative CPM scores indicate pain inhibition and positive CPM scores indicate pain facilitation. Efficient CPM was defined as the ability of the individuals to inhibit at least 29% of pain.^35^

### 2.4. Questionnaires

The following 3 questionnaires were completed when the participants came to the investigation.

#### 2.4.1. Quality of life

Quality of life was measured at the start of the study with the 36-Item Short-Form Health Survey (RAND-36), a health survey that consists of 8 concepts investigating physical and mental status.^20^

#### 2.4.2. Depression and anxiety

The Hospital Anxiety and Depression Scale is a psychometric questionnaire specifically developed for nonpsychiatric patients to identify the grade of anxiety disorder or depression. It consists of 2 subscales, anxiety and depression. The total score for each subscale was calculated as the sum of the respective 7 items (ranging from 0 to 21), with values being 0–7 for normal cases, 8–10 for borderline cases, and 11–21 for abnormal cases.^6^

#### 2.4.3. QuickDASH (Disabilities of the Arm, Shoulder, and Hand)

QuickDASH is a short, reliable, and valid measure of physical function and symptoms related to upper limb musculoskeletal disorders by shortening the full, 30-item DASH (Disabilities of the Arm, Shoulder, and Hand) Outcome Measure.^2^

## 3. Statistics

All statistical analyses were performed with IBM SPSS Statistic version 19.0.0.1, GraphPad Prism 8 and SAS version 9.4 (SAS Institute, Inc). The level of significance was set at a *P*-value <0.05. Descriptive data are presented as mean values and SDs or median with interquartile range [IQR 25–75]. Sex differences in pain report and questionnaire measures were compared with the Student *t* test or Mann–Whitney *U* test. As this cohort study aimed to recruit the maximum available subjects, no a priori power calculation was performed. To compare CPM between men and women with neuropathic pain and without pain and to compare differences between the subjects in the same group, a two-way analysis of variance (group and side) was performed. A post hoc unpaired *t* test was performed for between-group comparisons and also a post hoc paired *t* test for within-group comparisons. For AUC_time off_ and Time off, we estimated proportional odds models (cumulative probability models using the link function) including the following variables: group (pain, no pain), age, sex, VAS MAX c, and duration in the cold-water bath. To assess correlation between pain adaptability and pain modulation, Spearman rank correlation test (two-tailed) was used.

## 4. Results

### 4.1. Demographic details and subjects characteristics

Demographic details and the characteristics for the subjects are presented in Table 1. Fifty-four women (30 with neuropathic pain) and 77 men (39 with neuropathic pain) were recruited between December 2018 and March 2020. There were no age, BMI, or ASA physical status differences between groups. Only 4 subjects with chronic pain used opioids. Both women and men with neuropathic pain had the same types of nerve injuries localized to digital, radial, median, and ulnar nerves and also multiple nerve lesions (*P* = 0.698). The main mechanisms of injury in both women and men were sharp laceration (70%) followed by avulsion (30%). No differences were observed between the experimental groups of men and women with pain or painless neuropathy at bedside examination. No differences were observed in the physical and mental component of RAND-36 (*P* > 0.05), arm disability (*P* < 0.05) measured with QuickDASH, and anxiety or depression scores measured with the Hospital Anxiety and Depression Scale questionnaire (Table 1).

**Table 1:** Sociodemographic and clinical characteristics of the subjects included in study.

		Female	Male	Group diff (F, M)	NP (F)	NP (M)	Sex diff (NP)	nP (F)	nP (M)	Sex diff (nP)
Age (y)	Median [IQR]	48 [24–63]	46 [25–67]	0.401	48 [20–60]	48 [22–64]	0.671	50 [25–60]	45 [24–54]	0.190
Gender	Female/male (N, % from all subjects)	54 (41%)	77 (58%)		30 (22%)	39 (29%)		24 (18%)	38 (29%)	
Pain duration (y)	Mean ± SD	4.3 ± 2.5	3.9 ± 2.7	0.327	3.2 ± 2.1	4.2 ± 2.7	0.136	4.5 ± 2.1	4.6 ± 2.2	0.135
BMI (kg/m^2^)	Median [IQR]	26.1 [23–26]	27.3 [23–29]	0.397	25.2 [21–30]	25.5 [23–27]	0.816	25.3 [22–25]	27.1 [24–29]	
ASA	(N, % from all subjects, %NP, % nP)			0.542			0.736			0.564
	I	35 (26%)	45 (34%)		20 (28%)	22 (31%)		15 (24%)	23 (37%)	
	II	15 (11%)	27 20%)		7 (10%)	15 (21%)		8 (12%)	12 (19%)	
	III	4 (3%)	5 (3%)		3 (4%)	2 (2%)		1 (1%)	3 (4%)	
Pain medication	(N, % from all subjects, %NP, %nP)			0.431			0.613			0.754
	Opioids	2 (1%)	2 (1%)		2 (2%)	2 (2%)		0	0	
	Tricyclics/duloxetine	4 (3%)	2 (1%)		4 (5%)	1 (1%)		0	1 (1%)	
	Gabapentinoids	4 (3%)	3 (2%)		4 (5%)	3 (4%)		0	0	
	Paracetamol	4 (3%)	4 (3%)		1 (1%)	3 (4%)		3 (4%)	1 (1%)	
	COX inhibitors	5 (3%)	5 (3%)		2 (2%)	3 (4%)		3 (4%)	2 (3%)	
Employment (n)	(N, % from all subjects, %NP, %nP)			0.854			0.746			0.467
	Employed	46 (35%)	53 (40%)		19 (27%)	25 (36%)		17 (27%)	28 (45%)	
	Retired	10 (7%)	14 (10%)		5 (7%)	6 (8%)		5 (8%)	8 (12%)	
	Unable to work	7 (5%)	8 (6%)		6 (8%)	7 (10%)		1 (1%)	1 (1%)	
	Other	1 (0.7%)	2 (1%)		1 (1%)	1 (1%)		0	1 (1%)	
Nerve injury (n)	(N, % from all subjects, %NP, %nP)			0.662			0.608			0.440
	Digital nerves (total, %from all subjects) injured digit site (ulnar, radial)	31 (23%) (14, 17)	42 (32%) (20,22)	0.092	17 (24%) (7,10)	20 (28%) (8, 12)	0.609	14 (22%) (10, 4)	22 (35%) (12, 10)	0.333
	Median	9 (6%)	12 (9%)		5 (7%)	4 (5%)		4 (6%)	8 (12%)	
	Ulnar	6 (4%)	5 (3%)		4 (5%)	3 (4%)		2 (3%)	2 (3%)	
	Radial	3 (2%)	5 (3%)		1 (1%)	2 (2%)		2 (3%)	3 (4%)	
	Multiple nerves	5 (3%)	13 (9%)		3 (4%)	13 (18%)		2 (3%)	0	
Mechanism of injury	Sharp laceration Crush/avulsion % From subgroup	38 (70%) 16 (30%)	56 (72%) 21 (28%)	0.633	22 (73%) 8 (27%)	36 (92%) 13 (8%)	0.553	16 (66%) 8 (34%)	20 (52%) 18 (48%)	0.231
Reoperation	(N, % from all subjects, %NP, %nP)	11 (8%)	4 (3%)	NS	7 (10%)	3 (4%)	NS	4 (6%)	1 (1%)	NS
Dominant hand (right)	Right	42 (32%)	56 (42%)	NS	29 (42%)	36 (52%)	NS	13 (20%)	20 (32%)	
Injury site (right)	Right	25 (19%)	27 (20%)	NS	13 (18%)	17 (24%)	NS	12 (19%)	10 (16%)	
Pain intensity (VAS 0–100 mm) (median, range)	Maximum last week	52 (0–80)	38 (0–70)	***0.031***	80 (35–100)	60 (0–100)	***0.029***	0	0	NS
	Minimum last week	11 (0–30)	10 (0–30)	0.782	10 (0–53)	20 (0–60)	0.862	0	0	
	Average last week	31 (21–75)	23 (10–73)	***0.042***	55 (0–100)	49 (20–57)	0.318	0	0	
	Current	42 (23–60)	34 (10–60)	***0.032***	27 (15–50)	26 (0–85)	0.983	0	0	
Other chronic pain	(N, % from all subjects, %NP, %nP)	24 (18%)	25 (19%)	0.420	12 (17%)	14 (29%)	>0.99	12 (19%)	11 (17%)	NS
	Joint pain	11 (8%)	16 (12%)		8 (11%)	13 (18%)		3 (4%)	3 (4%)	
	Low back pain	8 (6%)	6 (4%)		1 (1%)	0		7 (11%)	6 (9%)	
	Headache	2 (1%)	2 (1%)		0	1 (1%)		2 (3%)	1 (1%)	
	Other	3 (2%)	1 (0.7%)		3 (4%)	0		0	1 (1%)	
LANSS part A	Median (range)	6 (9–16)	6 (3–6)	0.987	11 (8–16)	10 (8–16)	0.978	0 (0–8)	2 (0–8)	NS
LANSS part B	Median (range)	6 (6–8)	6 (3–7)	0.559	8 (8–8)	8 (8–8)	0.558	4.5 (0–8)	3.5(0–8)	NS
Bedside examination	(N, % from all subjects, %NP, %nP)			0.354			0.648			0.872
Loss of function	Touch	35 (26%)	37 (28%)		16 (23%)	18 (26%)		19 (30%)	19 (30%)	
	Pinprick	45 (34%)	46 (35%)		18 (26%)	28 (40%)		17 (27%)	18 (29%)	
	Warm	40 (30%)	38 (29%)		18 (26%)	22 (31%)		22 (35%)	16 (25%)	
	Cold	33 (25%)	29 (22%)		17 (24%)	16 (23%)		16 (25%)	13 (20%)	
Gain of function	Touch	48 (36%)	54 (41%)		24 (34%)	29 (42%)		24 (38%)	25 (40%)	
	Pinprick	25 (19%)	25 (19%)		10 (14%)	15 (21%)		15 (24%)	10 (16%)	
	Warm	25 (19%)	24 (18%)		11 (17%)	13 (18%)		14 (22%)	11 (17%)	
	Cold	33 (25%)	35 (26%)		14 (20%)	19 (27%)		19 (30%)	16 (25%)	
HADS anxiety	(N, % from all subjects, %NP, %nP)			0.133			0.992			
0–7	No anxiety	36 (27%)	60 (45%)		16 (23%)	29 (49%)		10 (16%)	31 (50%)	
8–10	Mild anxiety	10 (7%)	13 (9%)		7 (10%)	7 (10%)		3 (4%)	6 (9%)	
≥11–21	Severe anxiety	8 (6%)	4 (3%)		7 (10%)	3 (4%)		1 (1%)	1 (1%)	
HADS depression	(N, % from all subjects, %NP, %nP)			0.143			0.749			0.675
0–7	No depression	44 (33%)	58 (44%)		24 (34%)	36 (52%)		20 (32%)	22 (35%)	
8–10	Mild depression	7 (5%)	12 (9%)		3 (4%)	2 (3%)		4 (6%)	10 (16%)	
≥11–21	Severe depression	3 (2%)	7 (5%)		3 (4%)	1 (1%)		0	6 (9%)	
QuickDASH	(mean ± SD)	24 ± 22	23 ± 12	0.561	37 ± 16	31 ± 26	0.097	8 ± 11	7 ± 10	0.946
RAND-36	(mean ± SD)									
	PF	80 ± 20	85 ± 17	0.234	74 ± 19	79 ± 21	0.240	86 ± 17	91 ± 17	0.340
	RP	68 ± 37	66 ± 29	0.567	61 ± 35	48 ± 39	0.264	81 ± 27	84 ± 30	0.274
	BP	64 ± 22	68 ± 23	0.673	51 ± 20	53 ± 24	0.900	81 ± 23	84 ± 23	0.203
	GH	67 ± 25	72 ± 19	0.149	64 ± 25	70 ± 25	0.207	72 ± 20	74 ± 18	0.277
Physical component RAND-36	259 ± 85	260 ± 69	0.234	250 ± 76	260 ± 86	0.283	320 ± 70	330 ± 60	0.383	
	MH	77 ± 21	79 ± 18	0.663	75 ± 21	77 ± 20	0.463	82 ± 15	77 ± 17	0.240
	RE	77 ± 38	73 ± 32	0.098	66 ± 36	63 ± 39	0.763	87 ± 27	79 ± 34	0.264
	SF	82 ± 26	82 ± 22	0.984	78 ± 24	77 ± 28	0.903	87 ± 23	86 ± 30	0.900
	VT	60 ± 24	64 ± 20	0.429	60 ± 24	63 ± 39	0.778	62 ± 19	63 ± 20	0.207
Mental health component RAND-36		285 ± 99	280 ± 81	0.195	279 ± 66	290 ± 50	0.783	300 ± 55	290 ± 54	0.483

Data presented as n (%), mean ± SD, or median [IQR 25th, 75th percentile]. *P* value <.05 was considered significant. Bold text indicates a statistically significant difference with a p-value less than 0.05.

NP, neuropathic pain; nP, neuropathy without pain; NS, nonsignificant; BMI, body mass index; ASA, The American Society of Anesthesiologists Physical Status classification system; Pain intensity, VAS, average weekly pain on a visual analogue scale (0–100; worst = 100); LANSS, The Leeds Assessment of Neuropathic Symptoms and Signs Pain Scale; HADS, Hospital Anxiety and Depression Scale (0–21, worst = 21 for each subscale); QuickDASH, a shortened Version of Disabilities of the Arm, Shoulder, and Hand score; RAND-36, 36-items measure of health-related quality of life; PF, physical function; RP, physical role/function; BP, body pain; GH, general health; MH, mental health; RE, emotional role/function; SF, social functioning; VT, vitality. Independent samples Mann–Whitney *U* test was used for the between-group comparisons.

### 4.2. Pain intensity

Female subjects recorded higher pain ratings compared with male subjects in relation with maximum clinical pain intensity (*P* = 0.031), average pain in the last week (*P* = 0.042), and current pain (*P* = 0.032). Women in the group with NP displayed greater maximum and average clinical pain intensity in comparison with men (*P* = 0.029 and *P* = 0.018, respectively) (Table 2).

**Table 2:** Differences in endogenous pain modulation between female (F) and male (M) participants.

	All F and M								F and M with NP							F and M without pain						
Gender	N Obs	Variable	Median (range)	CI 95% lower	CI 95% higher	Mean	SD	Diff	N Obs	Median (range)	CI 95% lower	CI 95% higher	Mean	SD	Sex diff	N Obs	Median	CI 05% lower	95% CI	Mean	SD	Diff
F	54	AUC_time off_	1663 (52–6352)	1468	2185	1826	1313	0.72	30	1942 (791–6532)	1516	2642	2079	1507	0.90	24	1518 (743–1981)	1105	1918	1511	961	0.88
M	77	AUC_time off_	1446 (148–9536)	1858	1638	1896	1628		39	1943 (148–9536)	1585	2583	2329	2018		38	1314 (249–4041)	1214	1875	1545	1046	
F	54	Time off (s)	29 (0.8–104)	25	36	31	20	0.43	30	36 (10–100)	27	45	36	23	0.89	24	21 (5–57)	28	66	24	14	0.60
M	77	Time off (s)	27 (3–281)	29	49	39	43		39	29 (28–54)	25	36	47	57		38	27 (25–36)	7	74	31	17	
F	54	AUC_1_	6468 (257–14831)	5870	7603	6736	3175	0.18	30	5817 (254/13831)	5246	7871	6549	3543	0.51	24	6797 (1419–11475)	5803	8090	6947	2709	0.48
M	77	AUC_1_	5644 (199–12126)	5220	6530	5905	3019		39	5983 (198–12125)	4862	6782	5824	2957		38	5320 (391–12084)	4973	7193	5988	3991	
F	54	AUC_2_	4734 (359–12783)	4119	5877	4998	3219	0.98	30	3306 (359–12783)	3365	3806	4612	3390	0.62	24	5410 (973–11399)	4187	6774	5481	3063	0.70
M	77	AUC_2_	4755 (0–12106)	4287	5629	4990	3098		39	4980 (0–10192)	3949	5741	4843	2773		38	4497 (25–12026)	4017	6230	5143	4431	
F	54	∆-AUC	−1908 (−7674/2905)	−2360	−1117	−1738	2278	**0.02**	30	−2088 (−7397/2394)	−2782	−1130	−1956	2212	**0.03**	24	−1443 (−7946/2906)	−2460	−2762	−1466	2377	**0.05**
M	77	∆-AUC	−724 (−6549/4895)	−1401	−495	−948	1982		39	−908 (−5814/4895)	−1779	−320	−1050	2218		38	−579 (−6459/7459)	−4118	−264	−846	1738	
F	54	CPM effect	−23 (-82–79)	−30	−14	−20	29	0.61	30	−27 (-43–0)	−36	−12	−24	31	0.33	24	−15 (−82/16)	−30	−9	−19	25	0.39
M	77	CPM effect	−19 (-100–58)	−27	−12	−22	33		39	−15 (-100–57)	−31	9	−20	33		38	−23 (−82/48)	−30	−8	−19	32	
F	54	VAS max1	67 (3.5–121)	54	68	71	28	**0.03**	30	70 (36–130)	50	72	67	29	**0.02**	24	64 (14–94)	51	70	68	22	0.19
M	77	VAS max1	49 (3.4–98)	45	56	51	24		39	52 (32–97)	35	50	52	63		38	47 (3–98)	41	57	49	25	
F	54	VAS max2	46 (6.3–119)	39	55	57	31	0.56	30	44 (6–118)	35	58	46	30	0.80	24	45 (11–97)	38	59	48	26	0.70
M	77	VAS max2	41 (0–93)	36	48	43	25		39	59 (28–58)	35	50	42	23		38	63 (20–93)	33	51	42	27	
F	54	VAS max CS	60 (20–132)	84	94	89	17	**0.04**	30	70 (22–100)	80	94	87	18	0.91	24	90 (40–100)	85	98	92	16	0.63
M	77	VAS max CS	54 (14–99)	78	88	80	21		39	64 (50–100)	81	93	80	17		38	87 (24–100)	71	87	79	23	
F	54	AUC CS	4182 (844–12920)	2758	5700	4592	2458	0.13	30	4492 (892–12920)	3975	6160	5057	2829	0.668	24	3814 (1579–7646)	3318	4703	4011	1621	0.95
M	77	AUC CS	4111 (748–11135)	3947	4766	4281	2135		39	4448 (1570–11135)	3853	5298	4575	2228		38	3972 (748–7519)	3315	4613	3979	2020	
F	54	Total duration CS	67 (19–159)	54	97	75	30	**0.04**	30	81 (19–159)	69	95	80	35	0.687	24	65 (29–117)	56	73	64	20	**0.005**
M	77	Total duration CS	83 (21–83)	69	99	87	45		39	82 (21–310)	74	112	82	26		38	84 (20–139)	70	87	79	26	
F	54	Duration bath	48 (11–74)	38	48	44	18	0.14	30	53 (11–73)	39	53	45	19	0.747	24	38 (13–66)	33	46	40	14	**0.01**
M	77	Duration bath	55 (69–99)	41	60	48	17		39	60 (13–51)	41	51	48	16		38	55 (9–66)	43	55	48	16	

AUC1, area under the curve test 1, AUC2, area under the curve test 2, ∆-AUC, AUC_2_ − AUC_1_, %CPM, [(AVG2 − AVG1)/AVG1 × 100], VAS max1, maximum visual analog scale test 1, Time off, time from maximum pain intensity to 0 after conditioning stimulus; AUC time off, area under the curve from maximum pain intensity over time until the subjects became pain free; VAS max CS, the maximum pain intensity on visual analog scale; AUC_CS_ area under the curve, pain ratings in time under CS; Duration CS (s), total time of conditioning stimulus; duration in bath, seconds for hand in bath. Bold text indicates a statistically significant difference with a p-value less than 0.05.

### 4.3. Factors influencing conditioned pain modulation effect

To examine the factors influencing the CPM effect, Spearman correlations were computed between time off, AUC time off, and duration in bath. Consistent with previous research, correlational analyses revealed no significant intercorrelations between measures of CPM effect with time off. Time off had no correlation with duration in bath (Spearman *R* = 0.115, *P* = 0.118) and VAS max CP (Spearman *R* = 0.009, *P* = 0.921) but was correlated with area under the curve of the after-sensation time (AUC CP, *P* < 0.0001) and total duration of CP (*P* < 0.0001).

### 4.4. Sex effects on pain modulation

**Figure 2.**
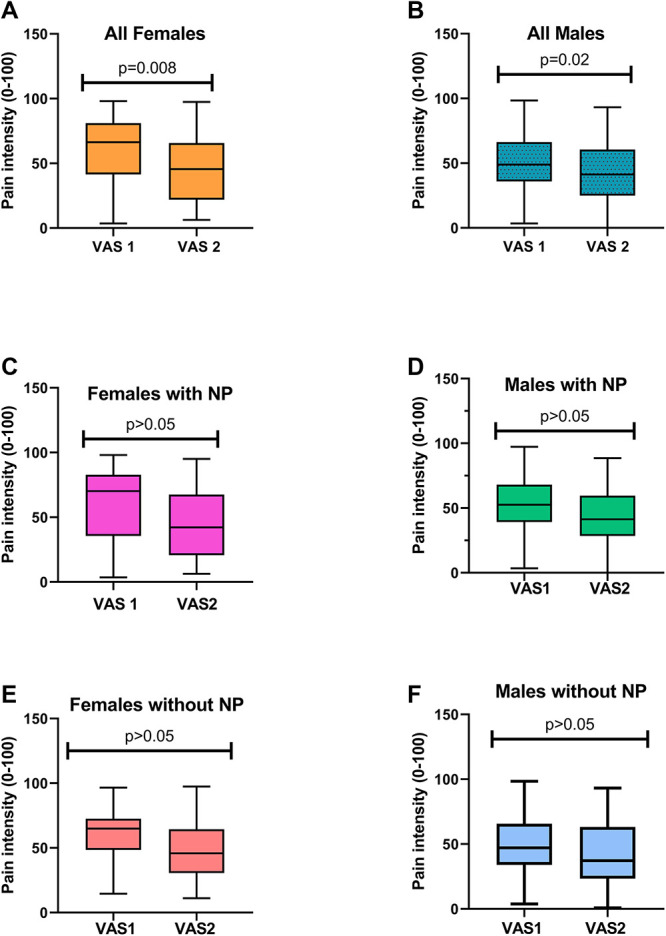
Box and whisker plots, with pain intensity expressed as VAS (0–100) during the first test stimulus (VAS1) and during the second test stimulus with before and after values for each subject. (A) In all women (orange), pain intensity ratings of VAS2 (VAS after test stimulus 2) were significantly lower than VAS 1 (VAS after test stimulus 1) (*P* = 0.008). (B) In all men (blue), VAS2 pain ratings were significantly lower than VAS1 pain (*P* = 0.02). (C, D, E, F) In the neuropathic pain subgroup of both female (pink) and male (green) participants and in subgroups without pain (red = female patients and blue = male patients), VAS2 pain ratings were not significantly different from VAS1 (*P* > 0.05). Error bars represent SD.

**Figure 3.**
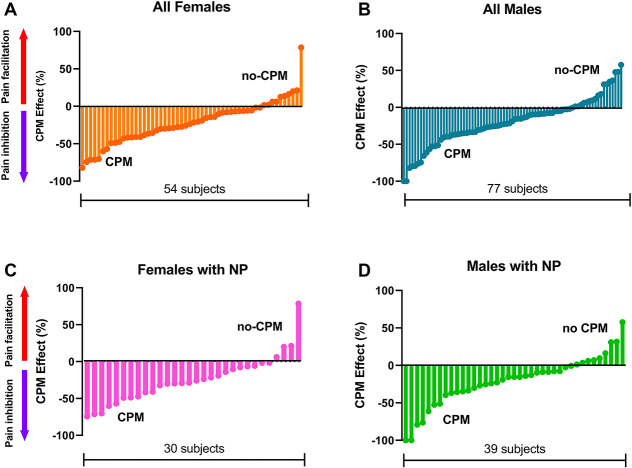
Individual pain inhibition or pain facilitation expressed as CPM effect percentage (%) change during the conditioned pain modulation paradigm in all female (A) and male participants (B). Distribution of CPM effect percentage (%) change in all female subjects with neuropathic pain (C) and all male participants with neuropathic pain (group D). Each bar represents the CPM effect percentage (%) change of a participant, and the participant number range is shown below each graph. CPM, conditioned pain modulation.

**Figure 4.**
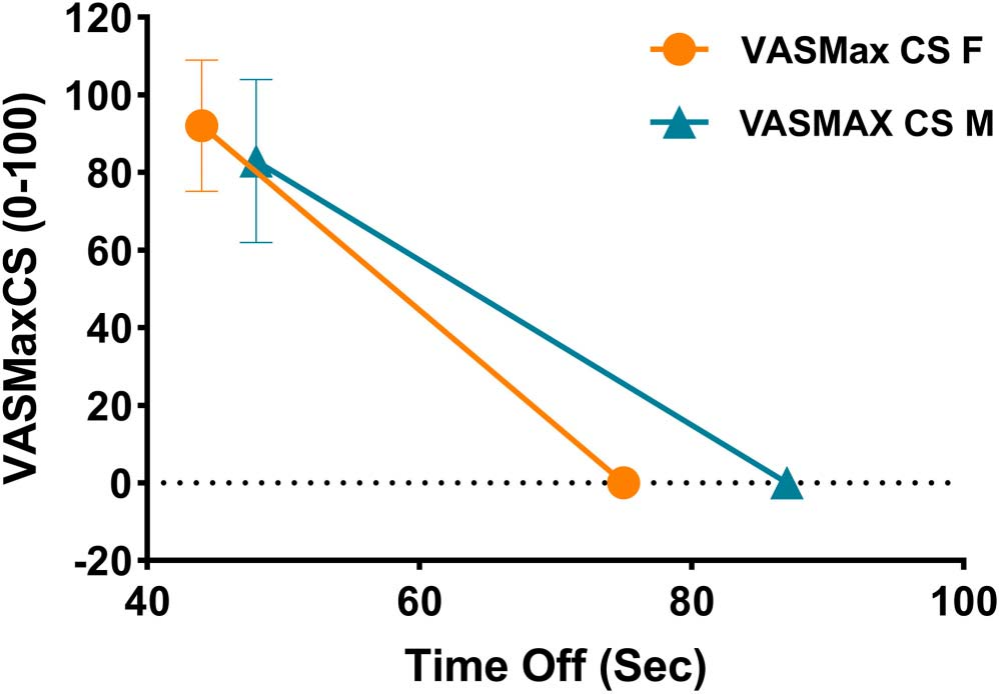
Time off for all female subjects (orange circles) and all male subjects (blue triangles). There are differences between VASMax CS (*P* = 0.04) between female and male subjects, but female subjects had less (20% of men) odds of recovering later.

There was significant difference in mean peak VAS during CS responsiveness between men and women (mean ± SD VAS_CS_ female: 89.5 ± 17.5, VAS_CS_ male: 83.5 ± 21.2, *P* = 0.04) (Table 2). Women rated experimental pain higher than men under the first test (Mann–Whitney *U* test, *P* = 0.03) (Fig. 2). Pain ratings were at the same level during the second test stimulus (VAS_max2_; *P* = 0.5) explaining an apparently more pronounced CPM (∆AUC) in women (*P* = 0.02). This finding was no longer significant after adjustment for VAS and no longer supported by the result that no differences were seen in the CPM effect (*P* = 0.61) (Fig. 3). After adjusting for age, sex, VAS MAX CS and duration in bath, there was no significant difference between men and women for either AUC Time off (*P* = 0.73) or Time off (*P* = 0.48). Summing all groups together, the odds ratio was 0.80 for Time off for women, interpreted as follows: the odds of a female patient recovering sooner after CS was 20% more the odds of a male patient (95% CI = 0.65–2.9) (Fig. 4). Time off had no correlation with duration in bath (Spearman *R* = 0.115, *P* = 0.118) and VAS max CP (Spearman *R* = 0.009, *P* = 0.921) but was correlated with area under the curve of the after-sensation time (AUC CP, *P* < 0.0001) and total duration of CP (*P* < 0.0001).

#### 4.4.1. Sex differences in the group with neuropathic pain

Women with neuropathic pain apparently had more CPM response in comparison with men (*P* = 0.03). This finding was no longer significant after adjustment for duration of test stimulus and VAS (*P* = 0.056). No statistically significant difference between male and female participants was seen in the group with NP for either AUC Time off (*P* = 0.90) or Time off (*P* = 0.89) (Mann–Whitney *U* test) (Table 2).

#### 4.4.2. Sex differences in the group without pain

In the group without pain, a longer duration in cold bath (*P* = 0.01) and a longer duration of CS was found for men (*P* = 0.005). There was no significant difference between male and female participants in the group without pain for either AUC Time off (*P* = 0.88) or Time off (*P* = 0.38) (Mann–Whitney *U* test). As in the other groups, differences in CPM were seen between male and female participants (*P* = 0.05), but this finding was no longer significant after adjustment for VAS (*P* = 0.06) (Table 2).

## 5. Discussion

The purpose of this study was to find potential sex differences in endogenous pain modulation, but contrary to our hypothesis, no sex differences in CPM were found. However, there was a difference in another parameter of the CPM measurements. A shorter after-sensation time after cold stimuli was found in female participants. In addition, this study provided insights into the differences between female and male participants demonstrating an increase in pain intensity after experimental pressure and cold stimuli in female participants without any relation to the presence of chronic neuropathic pain. No other contributing factors, such as depression and anxiety, life quality, or arm disability, have been related to a clear pattern of sex differences.

### 5.1. Population

Although the risk of developing neuropathic pain is known to be greater in female participants,^27,42^ the presence of chronic pain after traumatic nerve injuries could not confirm this general observation. Patients with peripheral nerve injuries with upper extremity involvement were more likely to be young male subjects in both severe trauma with multiple injuries^22^ (78.6%) or localized hand and arm injuries (between 66.5%–74% and 80%).^30,41^

### 5.2. Sex-dependent effects on pain modulation chronic neuropathic pain

In a previous study,^30^ we could not find a statistically significant interaction between the different sexes and chronic pain after traumatic nerve injuries in the upper extremities. In the present study also, contrary to our hypotheses, we did not find consistent differences between subjects with chronic neuropathic pain and neuropathy without pain. Similarly, the present study diverged from the general tendency that previously has indicated reduced CPM in female participants.^34^ The hypothesis that chronic neuropathic pain after traumatic nerve injuries is maintained by dysfunction in CPM has not received any support in this study. The time from injury and the pain duration was quite long and possibly the CPM results would have been different in the acute phase. Granosky et al. indicated that neuropathic pain duration significantly correlated with CPM efficiency and demonstrated that longer duration of diabetic neuropathy has been associated with more efficient CPM.^14^ Thus, the patients with neuropathic pain seemed to have “normalized” CPM with the chronicity of the pain syndrome.^14^ The same author found more efficient CPM among patients with painful diabetic neuropathy in comparison with those patients with painless diabetic neuropathy, which might result from altered sensory messages coming from tested affected body sites.^15^ Cold inhibition has been found greatest during the menstrual phases when comparing the ovulatory phase to the menstrual and luteal phases.^47^ It is difficult to draw any conclusion concerning the role of hormones in CPM because another study could not determine any variation of CPM during the menstrual cycle.^53^ With the limitation that we did not take any blood test to ensure the hormone levels, this study indicated that the CPM response in painful neuropathies was not related to sex differences. These results were similar to other studies with CPM in healthy individuals.^23,44^ Although female participants did not respond with an increase in magnitude of CPM, the results indicated they are predisposed to returning more quickly to baseline in comparison with male participants. Time off is an indicator of endogenous of pain modulation supported by distinct brain mechanisms in comparison with the CPM paradigm.^21,33^ In previous studies, a different response and functional involvement of the central nervous system to the same stimulus by male and female participants has been shown with functional MR^24,28^ or positron emission tomography.^25^ Significantly, more regional activation of the μ-opioid system in response to sustained pain was detected with brain imaging in male participants, suggesting that pain-induced endogenous opioid analgesia was significantly greater in male participants than in female participants.^54^ This would contradict our result that indicated a tendency to longer time off after sensation in male participant. However, in another study, experimentally induced discouragement triggered significantly more opioid-mediated analgesia in female patients but not in male patients.^12^ In patients with chronic pain, new cerebral pain-related associations and disruptions in pain-related cerebral activation occur that might influence endogenous pain modulation.^43^

### 5.3. Sex-dependent effects on conditioned pain modulation paradigm

There is evidence of difference in the magnitude of CPM related to sex that depend on the nature of the CPM paradigm.^19^ The method of delivering test pain and conditioning stimulus influenced the CPM results in men and women.^19,26^ Women show a greater adaptation to pain than men with respect to prolonged noxious stimuli^18^ specially to sustained heat pain.^17^ Therefore, current assumptions of women as being more pain sensitive than men do not apply to responses observed with sustained stimuli. CPM was a more stable measure for female than male patients using musculoskeletal pain models.^50^ Moreover, sex differences only exist for some types of pain measures such as lower pressure/thermal pain thresholds and pain tolerance in women. Only few studies showed sex differences for other types of pain thresholds, such as cold pain thresholds, or for chemical or ischemic pain tolerance.^37,38^

### 5.4. Sex-dependent effects on pain intensity

During the first test stimulus, female patients reported higher pain intensity ratings to experimental noxious stimulus compared with male patients. This indicated an increased perception of pain in women, known to be a result of biopsychosocial and anatomical differences in female patients compared with male patients.^4,32^ Lower pain tolerance and a lower threshold of pain detection were attributed to greater nerve density in women explaining why women feel pain more severely than men.^32^ Other studies demonstrated sex differences only for pain thresholds arguing that the differences were unlikely to be because of peripheral factors such as innervation density.^45^ Differences in experimental pain, with greater sensitivity to multiple pain modalities in female patients compared with male patients, have been found in previous studies.^4,40^ Although male patients responded with an increase tolerance to the cold pressor task, after adjusting with duration in bath and VAS, no differences between sexes in the time of after sensation were found. Interestingly, female patients had increased pain intensity but seemed to recover more rapidly than male patients after experimental cold stimuli. This was probably because of differences in central processing of pain^9^ and in the more effective mechanisms to modulate and cope with pain in women than men over time.^17^ The results are confounding and might be related to the complex nature of pain itself: female patients had lower pain thresholds but they could cope better with pain and returned at baseline sooner than male patients after a pain stimulus.

## 6. Limitations

There are study limitations to consider. The findings resulted in this study were likely to be influenced by methodological factors such as selection biases regarding the proportion of men and women because of patients' recruitment. CPM data were compared between subjects with neuropathic pain and neuropathy without pain after nerve injuries but not with an independent healthy control group without nerve injury. Peripheral nerve injuries treated with nerve reconstructions have a better prognosis than similar lesions without repair and probably other types of neuropathic pain. Possibly, the selected setting here to examine sex differences, compensating mechanisms, and related gender differences in this sample of subjects operated in Hand Surgery could be different from patients recruited from a pain clinic. In addition, sex hormones likely influence CPM response but we assumed a random distribution of menstrual phases in the women participating. Further work is needed to understand factors that contribute to the differences between male and female patients in the recovery after a painful stimulus and in the development of chronic neuropathic pain.

## 7. Conclusions

Despite the fact that no sex difference in the magnitude of CPM effect was identified, our study strongly suggests that men and women differ in their response to pain because of increased pain sensitivities to both pressure and cold stimuli and a tendency of faster return to baseline after cold pressor test for female patients. In conclusion, with all higher pain intensities, women showed a greater adaptation to the pain resulted from cold conditioning stimuli.

## Disclosures

The authors have no conflicts of interest to declare.
